# Best practices in intercultural health: five case studies in Latin America

**DOI:** 10.1186/1746-4269-3-31

**Published:** 2007-09-05

**Authors:** Javier Mignone, Judith Bartlett, John O'Neil, Treena Orchard

**Affiliations:** 1 Department of Family Social Sciences, Faculty of Human Ecology, University of Manitoba, 307 Human Ecology Bldg., Winnipeg, Manitoba, R3T 2N2, Canada; 2 Department of Community Health Sciences, Faculty of Medicine, University of Manitoba, 715 McDermot Ave, Winnipeg, Manitoba, R3E 3P4, Canada; 3 Faculty of Health Sciences, Simon Fraser University, 888 University Drive, Burnaby, British Columbia, V5A 1S6, Canada; 4 British Columbia Centre for Excellence in HIV/AIDS, Canada

## Abstract

The practice of integrating western and traditional indigenous medicine is fast becoming an accepted and more widely used approach in health care systems throughout the world. However, debates about intercultural health approaches have raised significant concerns. This paper reports findings of five case studies on intercultural health in Chile, Colombia, Ecuador, Guatemala, and Suriname. It presents summary information on each case study, comparatively analyzes the initiatives following four main analytical themes, and examines the case studies against a series of the best practice criteria.

## Background

The practice of integrating western and traditional indigenous medicine is fast becoming an accepted and more widely used approach in health care systems throughout the world [1]. However, debates about intercultural health approaches have raised significant concerns regarding regulation, efficacy, effectiveness, intellectual property rights, lack of cross-cultural research, access and affordability, and protection of sacred indigenous plants and knowledge [2]. Further, the practice of integrating both systems is progressively taking place in correspondence with increased organization among indigenous communities and the development of their own health services [3].

Intercultural health in this paper is understood as practices in health care that bridge indigenous medicine and western medicine, where both are considered as complementary. The basic premises are that of mutual respect, equal recognition of knowledge, willingness to interact, and flexibility to change as a result of these interactions. Intercultural health takes place at different levels including that of the family, practitioner, health centre, hospital, and health system. A "best practice" in health care needs to satisfy a series of criteria. It should demonstrate a tangible and positive ***impact ***on the individuals and population served; be ***sustainable***; be ***responsive and relevant ***to patient and community health needs and to cultural and environmental realities; be ***client focused ***including gender and social inclusion; ***improve access***; ***coordinate and integrate ***services; be ***efficient and flexible***; demonstrate ***leadership***; be ***innovative***; show potential for ***replication***; ***identify health and policy needs***; and have the ***capacity for evaluation ***[4].

This paper reports on the findings of five case studies on intercultural health in Chile, Colombia, Ecuador, Guatemala, and Suriname, conducted by the First Nations Centre for Aboriginal Health Research at the University of Manitoba with the assistance of consultants in each of the countries (most of whom were indigenous). The study was funded by the Inter-American Development Bank (IDB) to provide evidence for program and policy development of socio-culturally appropriate solutions to increase availability and quality of services in health among indigenous peoples in the Americas. At the proposal stage a study framework was developed by the research team to assess initiatives of intercultural health against the above mentioned best practice criteria and to comparatively analyze the cases across four common analytical themes: Cultural, financial and management approaches to intercultural health service development; Opportunities and benefits provided by the intercultural health initiatives; Constraints and risks associated with the articulation of indigenous and western health systems; Assessment of impacts of intercultural health system development. The themes were developed to articulate broader health system domains when analytically comparing the intercultural initiatives. They add a level on inquiry to the understanding of how well best practice criteria were met in the different case studies. The best practice criteria utilized were derived from a study conducted by the National Aboriginal Health Organization of Canada that had developed a framework of best practices for aboriginal health and health care [4].

## Methods

This study used a replicative case study design, which is widely utilized in anthropological research, and is also employed where the focus is on a holistic understanding of how and why certain events or decisions have occurred over time [5]. "Replicative" refers to the replication of similar methodology in separate cases that enables a series of comparative analyses. Case study design has been defined as "an empirical inquiry that: a) investigates a contemporary phenomenon within its real-life context, b) the boundaries between the phenomenon and context are not clearly evident, and c) multiple sources of evidence are used." [5] Case study methodology must meet the scientific standards of validity and reliability. Validity was satisfied in our study by using multiple sources of evidence, maintaining an accurate and transparent record of the data collection process, and providing for the participation of case study stakeholders in the analytical process. Having multiple investigators examine the specifics of each case and to compare observations also strengthened validity. Rigorous documentation of the data collection process across case studies and investigators increased reliability.

The cases were chosen in consultation with the IDB and the Pan-American Health Organization (PAHO), exemplifying ongoing intercultural health initiatives with differential organizational structures and background. Data collection took place between August 2004 and January 2005, with an average of eight days of fieldwork in each country by the Canadian researchers, accompanied by the local consultants. Additionally, these consultants conducted prior in-country work to produce background documents in advance of the fieldwork, and organized the extensive, and often complex and sensitive, fieldwork logistics. The study proposal was approved by the Research Ethics Board of The University of Manitoba.

Across the five case studies, the research team interviewed a total of 69 individuals on a one-to one basis and conducted 56 group interviews and meetings (Table 1). Among these participants were indigenous community members, health systems users, western and traditional health care providers, health administrators, indigenous organizations and community leaders, NGO staff, multi-level and sector government officials, and IDB and PAHO country officials. The researchers also participated in community events that in total included approximately 450 people. These events included a community ceremony for a *Machi *(traditional healer) in a rural area near Temuco (Chile), a village celebration and feast in the Cauca region (Colombia), and a village assembly near Otavalo (Ecuador). At all sites, after initial orientation with consultants, data gathering began at the local level and then moved to regional and finally national levels. Within the local level, data was first gathered from indigenous service providers and community members, next from indigenous political organizations, and finally from government and organizations or western health systems. This allowed for better contextualization of interviews related to government policy such that they could be analyzed through a 'community lens'.

**Table 1:** Summary Information of each Case Study

	**Suriname**	**Guatemala**	**Chile**	**Ecuador**	**Colombia**
**Case Study**	Medical Mission & Amazon Conservation Team clinics in Trio villages.	Comadronas (Midwives) Association in Comalapa, Kaslen Foundation, health center	Makewe Pelale Hospital, Boroa Health Centre, Mapuche Pharmacy	Jambi Huasi Clinic/Midwife Association/Yachac Association	Consejo Regional Indígena del Cauca/Asociación Indígena del Cauca/Instituciones Prestadores de Servicios de Salud
**Place**	Kwamalasamutu & Pëlele Tëpu	Comalapa & surrounding areas	Temuco & surrounding areas	Otavalo & surrounding areas	Popayan & other areas in Cauca region
**Description of Initiatives**	-Western medical clinic & Traditional Shaman's clinic operate independently in remote indigenous villages. -Joint collaborations: workshops, mutual referrals, etc.	-Comadronas association supported by a local health promotion NGO (Kaslen) provide approximately 85% of childbirth services to Mayan women in remote areas -Comadronas receive training from government health centre	-Makewe Hospital and Boroa Health Centre run by Mapuche indigenous organizations offer both western medical services and traditional services with funding for western services provided by national government. Indigenous services are supported by both patients and administrative savings. -Mapuche operated Pharmacy in Temuco sells traditional medicines	-Jambi Huasi Clinic provides western and indigenous health services simultaneously in private fee-for service clinic. Fees for both western and indigenous healers are modest and identical. Patients select appropriate service and cross-referrals occur regularly. -Collaborates with Indigenous Midwife Association -Collaborates with Yachac's (traditional healers) Association	-Health Insurance Company (owned and operated by the indigenous regional council) enrolls indigenous clients and purchases indigenous and western services on their behalf from Health services providers mostly owned and operated by indigenous organizations.
**Individual Interviews**	10	10	7	12	10
**Group Interviews /Meetings**	12	7	11	14	12
**Total participants**	73	57	39	96	93
**Community Events**	None	None	1 (250 people)	1 (50 people)	1 (150 people)
**Locations Observed**	8	9	7	5	12
**Documents/data files**	12	12	42	25	23

Data collection was achieved through open-ended and semi-structured key informant interviews as well as structured interviews. In general, key informants in each case study were identified in consultation with country consultants. Interview guides were pre-designed seeking to operationalize the research questions. Nonetheless, the style of interview allowed for flexibility in the topics covered. Extensive notes by at least two researchers were taken during the interviews and later transcribed into computer files. Where necessary, interviews were conducted in the relevant indigenous language with the assistance of interpreters. Oral consent to participate in the study was obtained from local communities, organizations, agencies, and individuals. Observations were recorded, and numerous written documents and some quantitative data were gathered and reviewed. The documents consisted mainly of printed information produced by the organizations themselves or of previous studies conducted by other researchers. The quantitative data was mostly utilization data gathered by the cases for operational purposes. The observations were recorded as rigorously as possible, given the specific context of each observational activity. Some activities were video recorded or photographed. In all instances documentation of observations did not include personal identifiers to protect the anonymity and confidentiality of case study participants. Analyses were completed following the pre-designed case study framework.

## Case Study Descriptions

### Suriname

The first case study was in the southern area of Suriname, mainly in the village of Kwamalasamutu, which is an interior Amazon locale that is a two-hour flight from the capital city of Paramaribo. One clinic provides western medical care, run by a local NGO Medical Mission and funded primarily by the government. The other clinic provides traditional indigenous medicine, is operated by elder tribal shamans of the village, and is mainly financed by the Amazon Conservation Team (ACT), a US-based NGO. This clinic is built adjacent to the health outpost and provides sufficient space for several healers to practice traditional medicine. Patient visits to the traditional medicine clinics remain entirely elective. A second traditional clinic visited in another village, Pëlele Tëpu, has the same characteristics.

Shamans, Medical Mission health workers and physicians, and ACT members lead workshops to raise awareness among primary care practitioners about traditional health practices, important medicinal plants, and indigenous concepts of health and illness. The workshops also train traditional healers on basic primary care issues and preventive health practices. As a result, both the primary care workers and the shamans have altered their practice. Depending on the diagnosis and type of treatment required, referrals are routinely made between the two clinics. With the goal of preventing the disappearance of traditional knowledge, there is a Shamans and Apprentices program that encourages young apprentices to learn from the elder shamans and to preserve the knowledge of medicines from the Amazon rainforest. Apprentices are also trained to complete record forms that document conditions and treatments utilized for each patient at the traditional medical clinics. Voucher specimens of medicinal plants utilized in the clinics are obtained for taxonomic determination.

### Guatemala

The Guatemalan case study was based in San Juan de Comalapa, a Municipality with 35,441 people that is divided between a town centre and 27 surrounding villages and hamlets. It is located 24 kms from the urban centre of Chimaltenango and 85 kms from Guatemala City. Comalapa's population consists almost entirely of Kaqchikel Mayas (95.2%).

This case study focused on the role of "*comadronas*" in the health care system. *Comadronas *provide midwifery care to approximately 85% of pregnant mothers in the Mayan community. These women have played an important cultural and empirical role in the healthcare system of Mayan communities for centuries. *Comadronas *are responsible for assisting with pregnancy and childbirth, and for providing spiritual guidance to mothers and families. Additionally, they administer spiritual and empirical treatments to infants with cultural illnesses (e.g., *susto*, *mal de ojo*).

The "best practice model" in this instance is to link *comadronas *with the professional health system through the development of a training programme that is intended to increase the quality of care provided by the women, and to provide them with the knowledge and skills necessary to know when to refer their clients to the professional healthcare system. The ultimate aim of this model is to extend coverage of the western medical system into the poorest and most remote Mayan villages.

Sixteen *comadronas *of Comalapa have recently formed a Midwives Association together with nearly 65 *comadronas *from surrounding villages, with the support of the Kaslen Foundation, a local Mayan NGO. Comalapa also has a health centre and seven health posts across the region. The nearest hospital is in Chimaltenago, which is located 25 kms away from Comalapa.

Training programs for *comadronas *have varied considerably over the past few years. Both the public health system and the Kaslen Foundation have initiated programs for these attendants at various times in the past few decades. The health centre also introduced training initiatives for *comadronas *in 2002. The women participate in a one-week program in pre-natal care and recognition of complications, at the end of which they receive a certificate that allows them to register births. Since registration of births is important for families, this permit acts as a license to provide midwifery services. *Comadronas *who do not complete the training are technically unlicensed to provide care, although this does not seem to limit the practice of unlicensed *comadronas*.

### Chile

In Chile, the study was conducted at several health facilities in the city of Temuco and nearby areas. Temuco is located 670 km south of Santiago and has a population of some 300,000. The rural Makewe Hospital is situated in the territory of Makewe-Pelale, a historical Mapuche territory 25 km south of Temuco in the municipalities of Padres Las Casas and Freire. The Boroa-Filulawen Health Care Centre is also located in historical Mapuche territory. It is an area with 55 communities within the municipality of Nueva Imperial, 45 kms from Temuco. The Mapuche Pharmacy and urban traditional clinic are located in the city of Temuco.

The intercultural program focuses primarily on building a system where the power of traditional medicine embodied in the *Machi *(traditional healer) is offered as an equal and complementary alternative to western medicine. This vision is strongly embedded in a context of self-determination, as the recovery of traditional medicine is directly linked to social, political, and economic development in the Mapuche communities. The first initiative undertaken in 1998 was the development of the Makewe Hospital intercultural program, owned and operated by an association of Mapuche leaders. This Association is accountable to a Council of Mapuche Community Presidents from communities in the surrounding Makewe region. The Makewe Hospital provides a range of western health services under the direction of a western-trained Mapuche medical director. These include full-time physician services that are supported by nurses and nurse auxiliaries, midwives, visiting specialists, a dental clinic, and a social work department. An intercultural health worker is on staff and patients are seen by a Mapuche staff member and a western physician to ensure that if the patient has health needs that can only be met by traditional medicine, they are referred appropriately. The hospital holds a medical ward with 35 beds, a polyclinic, and a waiting room with a reception. The Mapuche Association is a not-for-profit corporation, and as such sells western health services to the government. Although linked to the work of the hospital, Mapuche medicine is not provided in the hospital, and *Machis *or other healers are not paid by the Association.

The second intercultural initiative was the development of a health centre in the community of Boroa, which was spearheaded by 25 Mapuche communities that did not have easy access to physicians and traditional services at the Makewe Hospital. The Boroa-Filulawen Health Care Centre has a *Machi *who attends the clinic one day per week but then treats patients at her home. Patients pay directly for her services similar to the system described above for the Makewe Hospital, although the health centre subsidizes the *Machi *with a small direct payment. Patients who have been diagnosed by either a western physician or a *Machi *have the choice of selecting herbal medicine instead of western medications, or as a complement to the latter.

A third component of the intercultural initiative are a traditional clinic and a pharmacy in Temuco directed by the Makewe Hospital Association.

### Ecuador

In Ecuador, the research was conducted predominantly in Otavalo, located in the province of Imbabura. The city of Otavalo is 110 km from the capital, Quito, and 25 km from the provincial capital Ibarra. The city has a population of almost 30,000. The official language is Spanish but many people also speak Runa Shimi or Kichua. The Otavalans are one of the most recognizable indigenous peoples in the Americas due to unique historical and socio-cultural dynamics that have permitted them to maintain their customs and traditions over time.

This case study describes the indigenous health program in the Otavalo region of Ecuador. The central component is the Jambi Huasi Clinic in the town of Otavalo. The clinic has been in operation since 1990 and provides a full range of western and indigenous health services to a population of 40,000 people on a fee-for-service basis. The Jambi Huasi Clinic operates under the authority of FICI (Federación de Indígenas y Campesinos de Imbabura), an indigenous organization supported by 160 communities in the region.

Jambi Huasi occupies an older house in a central area of the town of Otavalo. The clinic offers western, indigenous, and alternative health services. Western services include physicians (two sharing a full-time position), a dentist that is available four days per week, and a laboratory that provides medical tests like Pap smears and HIV/AIDS tests. Indigenous services include a *Yachac *(Spiritual Healer available one day per week), a *Fregadora *(Herbalist/Massager on a full-time basis) and a Midwife (full-time). One alternative practitioner specializing in Chinese massage and acupressure is available full-time as well. The clinic also has a health promotion facility that integrates knowledge from western and indigenous systems.

The second component is the Indigenous Midwives Association. This Association was formed in 2002 to monitor the certification of traditional midwives (*Parteras*) in the region. There are 64 midwives registered with the Association, although there are many more in the Otavalo region. The third component of the case study is the Yachac Association of Iluman, a small community near Otavalo. *Yachacs *are the spiritual healers in the Kichua community and there are a large number of them concentrated in this region due to the sacred nature of the area. In 2005 there were 47 members of the Association, including members from surrounding localities. Although the Yachac Association operates independently of all other health services and organizations, Jambi Huasi advocates with government and the western health care system on their behalf.

### Colombia

This case study was based in the department of Cauca in southwestern Colombia, which has a total population of 1,276,423, of which 190,069 are indigenous. Contrary to the general tendency in Colombia, the majority of the population in Cauca lives in the rural area (65%). It is a department with little economic growth, and agriculture is the major economic activity, followed by agricultural manufactures, and commerce. There are 81 indigenous "resguardos" (reserves) in Cauca, 108 indigenous Cabildos (local indigenous governments), and 10 Cabildo associations.

In 1993 the health reform in Colombia culminated with Law 100 that created the social security system. The essence of the system reform was the provision of coverage to persons under contributory and subsidized regimens that are based on a partnership scheme of income redistribution, which ensures universal benefits through the protection of the insured and family members. The subsidized regimen covers the most vulnerable population. Entities called "health promotion enterprises" were created and are responsible for the financial resources, health promotion, and organization and delivery of medical services. They are in essence health insurance schemes.

In 1997 the Consejo Regional Indigena del Cauca (CRIC) created a health insurance company, the Asociación Indígena del Cauca (AIC). Funded under the subsidized regimen it currently has 166,000 members. Nonetheless, differential indigenous population estimates suggest that up to 40% of the indigenous people in the CRIC region are not yet members of AIC. AIC staff is 70% indigenous and at the management level 100% of the staff is indigenous. At the operational level (delivery of care and programs) staffing is 60% indigenous and 40% non-indigenous. The model includes both indigenous governance and institutions, and the main articulating organization is the CRIC.

The AIC is a special character public entity constituted by 102 indigenous authorities, whose objective is to administer the health subsidies. It is directed by an administrative *junta *chosen by the General Assembly of Cabildos of the AIC and is presided over by a legal representative also chosen by the Assembly. Community involvement is ensured through designations at a local level and at a regional level through the participation in debates and decisions at community assemblies, "vereda" meetings, zonal assemblies, regional directive boards, directive boards of the AIC, and the Congress of CRIC. The community is not simply a user of the system, but through CRIC, AIC and the IPSs (Institutional Health Service Providers) it takes part in the decision-making process directing the health system.

The model is financed through public monies in the form of contracts between the municipalities and AIC. The model is also supported through its own resources that originate from the General System of Participation, which the indigenous reserves have a right to, and from international cooperation funds of CRIC's health program.

## Results

### Comparative analysis

This section comparatively analyzes the five cases following the four main analytical themes of the study: 1) cultural, financial and management approaches to intercultural health service development; 2) opportunities and benefits provided by the intercultural health initiatives; 3) constraints and risks associated with the articulation of indigenous and western health systems; 4) and assessment of impacts of intercultural health system development.

### Cultural, Financial and Management Approaches to Intercultural Health Service Development

The notion of interculturality has different expressions across the case studies. Suriname is relatively clear-cut with two clinics, a western and a traditional, interacting in indigenous villages. The informal collaboration between these entities enhanced the work of each and has garnered significant community support. The case studies in Chile, Ecuador and Colombia are western health care organizations offering intercultural health care services, although each attempts this in somewhat different ways. The initiative in Guatemala attempts to articulate a western health care public institution with indigenous organizations in the area of midwifery.

The governance and management models of the five cases parallel the above description. While the Suriname and Guatemala experiences have one entity in charge of western medicine and another for traditional medicine, the other three initiatives have indigenous entities managing both aspects. However, in all cases there are attempts at articulation of cultural approaches within the broader health system at different levels. Unfortunately it would appear that this goal is seldom realized and there is considerable evidence that racism is institutionalized in hospitals and other sectors of the health care system.

In terms of resources, the main funding for the traditional indigenous medicine aspect of the initiatives mostly came from non-governmental donors, fee-for service, or re-allocation of surplus administrative funds. Rarely does government provide any direct funding for indigenous traditional health services. Given the limited and insecure basis for this funding, the indigenous governance and management of the health delivery entities strengthened the integration of indigenous health services into the health care system.

### Opportunities and Benefits Provided by the Intercultural Health Initiatives

The case studies suggested a number of interesting opportunities provided by intercultural health initiatives. Opportunity for exchanging knowledge between both types of practitioners was particularly visible in Suriname and Ecuador, and to a somewhat lesser extent in Chile and Colombia. Despite efforts in Guatemala, the model emphasized western practitioners "training" the *comadronas *instead of a two-way exchange, and this approach constrained opportunities for knowledge exchange.

Another significant opportunity was an increase in trust among community members towards the health care system. Community trust of both the western and traditional clinics in Kwamalasamutu, Suriname, originated from a positive experience with each clinic separately, but also seemed to be reinforced by the collaboration between the two. On the other hand, in the Guatemala experience, the lack of trust between the *comadronas *and the western health centre has hindered the development of trust in intercultural work.

In Chile, the political mobilization of Mapuche communities has strengthened their position and enabled them to improve access to both western and traditional medicine. As well in Cauca, Colombia, indigenous run health insurance and health services companies provided powerful opportunities for indigenous governance and management of health care. In Otavalo, Ecuador, the Jambi Huasi experience has proved valuable in educating western health care staff to indigenous health and facilitating communication and trust with indigenous communities.

The benefits can be summarized as an apparent increase in cultural pride among the indigenous communities, although the situation in Guatemala is more ambivalent. Overall, the revaluing of traditional knowledge and practices and the increased sense of ownership and control over the health system appear to provide a wide range of potential benefits to indigenous communities.

More specifically, the articulation of indigenous and western systems seemed to facilitate more timely and appropriate referrals when medical care of higher complexity is required. In Otavalo, Ecuador, the collaboration of the municipal government with Jambi Huasi and the midwives association has enhanced initiatives of maternal, child and adolescent health. In Cauca, Colombia, the success of the indigenous health insurance company has increased the respect towards indigenous governance and management of health systems. In Temuco, the Makewe initiative has created a new dialogue around the value and role of traditional medicine and the responsibility of indigenous leadership in health issues.

### Constraints and Risks Associated with the Articulation of Indigenous and Western Health Systems

One set of constraints on intercultural health initiatives was related to the resistance from certain churches to traditional medicine or aspects of it. In Suriname, this was evidenced in the sidelining of the ceremonial spiritual practices of Shamans, not the use of traditional medicines per se. In the other cases, the resistance by mostly evangelical Christian churches was at times more overt. However, these contraints based on religious beliefs do not seem to have seriously limited any of the intercultural health iniatives studied.

Constraints related to health professionals differed across cases. In Suriname doctors and nurses working in remote areas were quite open to collaboration with traditional medicine. In Guatemala western health professionals indicated a degree of acceptance, but also felt that traditional practitioners should work as adjuncts to the western system. In Chile there was evidence that recent medical graduates are interested in practicing in indigenous settings precisely because of the experience of interculturality. In Ecuador integration between western and traditional practitioners works well in a specialized intercultural clinic, but there was little support for indigenous medicine among western health professionals in general.

In all cases, the relationship with personnel at the hospital level was not particularly positive, thus limiting the cultural appropriateness of services. The lack of clarity in relation to the legal framework for the practice of traditional medicine, and its interaction with western medicine, also creates many constraints. The legal situation in Chile is not currently of concern, but ambiguity in the legal codes places the experiences at risk if the government's position changes. Even in Colombia, where important legislation provides reasonable legal backing for intercultural initiatives, the lack of proper regulations supporting an integrated system constrain further developments. Ecuador constitutionally protects traditional healers, but lacks clear regulations as to how the public health system can interact with them. In Guatemala, the legislative situation of *comadronas *is unclear, although the public health system seeks to both regulate them through a registration system and entice them to receive training. In Suriname, the lack of a regulatory framework does not seem to have constrained the intercultural initiatives.

The lack of secure funding engenders many constraints across the five cases. Particularly in Guatemala, a very limited state health budget has prevented any broad articulation between the *comadronas *and the health system. The experience in Colombia indicates that even where financial resources are scarce, indigenous health resource governance and management enhances the viability of intercultural health. In Suriname, without support from a foreign NGO, the traditional clinics would not likely be operating. The Makewe Hospital in Chile has government funding support for western health services, and administrative efficiencies provide a small amount of funding for the intercultural experience. The Jambi Huasi Clinic in Ecuador is very efficient with financial resources provided by international NGO's, but these have not been continuous and the clinic is forced to rely on fee-for service income (albeit at a modest level) to survive.

The potential risk of iatrogenic consequences regardless of the system of medicine followed was identified by key informants in most case studies. A common theme was the acknowledgement of increased risk when a proper articulation across the two systems is lacking. The cases of Ecuador, Chile, Suriname and Colombia suggested intercultural models with certain level of monitoring within and across the two health systems seeking to minimize potential iatrogenic risks.

Finally, the lack of adequate data collection systems in all cases, Suriname being somewhat of an exception, seriously constrains these intercultural health initiatives in terms of planning, operations, monitoring, evaluation and research.

### Assessment of Impacts of Intercultural Health System Development

The most likely impact in four of the five cases of the intercultural health initiatives was an apparent increase in access to both traditional and western medicine. Guatemala was an exception because the intercultural model did not seem to be functioning properly. The cases suggested that when indigenous entities are involved in organizing health care there was an impact in reducing barriers to access and increasing user satisfaction.

The cost of traditional medicines and practitioners vis á vis western medicine is minor. The case studies revealed that the systems do not function in opposition to each other. On the contrary, the real choice is between investing in inclusive intercultural health care models instead of systems based only on western health care.

A positive impact of most intercultural health initiatives was in relation to indigenous community development, including revalorization of indigenous knowledge, cultural continuity and pride as a people. The initiatives not only seemed to positively impact health care, but also the development of community participation and organization, which had an impact on broader health determinants such as nutrition and employment.

The case studies suggested the plausibility of these initiatives in improving access, satisfaction, increasing treatment options in health care, impacting broader health determinants, and consequently having a positive effect on health status. Nonetheless, a different study design and adequate data collection systems would be required to provide evidence of the effectiveness of these intercultural health systems. There was a lack of adequate information systems in the five cases for both the western health care practices and the traditional indigenous practices. The development of appropriate information systems would surely be of assistance to the intercultural health initiatives.

### Case study initiatives compared against the best practice criteria

Table 2 summarizes our findings in comparing case studies against the best practice criteria based on three levels of achievement, criterion *met*, *partially met*, and *not met*. Granted, there is an element of relativity in this categorization. The assessment of *met *refers to evidence suggesting a sufficient level of achievement of that particular criterion as defined in the framework. When the level of achievement was limited the criterion was categorized as *partially met*. When there was evidence of not having achieved basic levels, it was assessed as *not met*. This assessment was conducted independently by each of the three main researchers using the evidence collected. In the few cases of discrepant ratings, the evidence was again reviewed and a consensus was reached. The Colombian and the Chilean experiences were the cases that met most of the criteria, followed by the initiatives in Suriname and Ecuador. The Guatemala case failed to meet most of the criteria.

**Table 2:** Five Case Study Initiatives Compared Against the Best Practice Criteria

**Project/Criteria**	Shaman's Clinic & MM Clinic (Suriname)	Comadronas Midwifery (Guatemala)	Makewe Hospital/Boroa (Chile)	Jambi Hausi/Midwife and Yachac Associations (Ecuador)	CRIC/AIC/IPS (Colombia)
**Impact**	pm	nm	pm	pm	pm
**Sustainability**	pm	nm	pm	pm	m
**Responsiveness and Relevant**	m	pm	m	pm	m
**Client Focus**	m	pm	m	m	m
**Access**	m	pm	m	m (urban)	m
**Coordination & Integration**	m	nm	m	pm	m
**Efficiency & Flexibility**	pm	pm	pm	pm	pm
**Leadership**	pm	pm	m	m	m
**Innovation**	m	pm	m	m	m
**Potential for Replication**	pm	pm	pm	pm	pm
**Health/Policy Identification or Resolution**	pm	pm	m	pm	m
**Capacity for Evaluation**	pm	nm	pm	nm	pm
**Criterion**					
**m = met**	5	0	7	4	8
**pm = partially met**	7	8	5	7	4
**nm = not met**	0	4	0	1	0

*Impact *refers to the notion that the initiative can demonstrate some tangible and positive health improvement on the individuals and population served or improvement for health care providers that can be measured quantitatively or qualitatively. In the absence of reliable quantitative data across the five cases in both the western and traditional medicine experiences it was impossible to determine impact at either the individual or population level. However, we were able to use qualitative information to reach a tentative assessment of positive impact in Suriname, Chile, Ecuador and Colombia. This was not clear in the case of Guatemala. The positive impact of the intercultural health initiatives was apparent at several levels including having strengthened indigenous organizations, cultural identity and continuity. The plausibility of improved health can be deduced insomuch that increased referrals to higher complexity care, more culturally appropriate primary care, preventive services and health promotion activities, and improved social/cultural determinants have a positive effect on health.

The Colombian and the Chilean experiences demonstrated a plan for viability and continuity of the initiatives through existing contractual arrangements with the state. The *sustainability *of the Suriname experience is positive organizationally and economically but externally dependent on funding. The Jambi Huasi Clinic in Ecuador has demonstrated that it is economically self-sustainable through fee-for-service funding but this has severely limited the reach of its programs. In Guatemala, both government and indigenous organizations have plans for sustainability but as yet, none have been realized.

A high level of *responsiveness *to patient and community health needs, as well as to cultural and environmental realities was shown in all five case studies. Despite very different origins, all five initiatives appeared to have emerged in response to needs of the indigenous communities for access to both health services and strengthening of cultural identity. The intercultural health initiatives in at least four of the five cases were the result of ongoing struggles by indigenous organizations that were able to take advantage of constitutional and legislative changes (these also partially a result of indigenous struggles) to develop systems that seek to respect and integrate western and traditional health models.

In line with responsiveness, all five initiatives demonstrated *client focus *in their sensitivity and provision of appropriate opportunities for individuals and communities, as well as special attention to elders, women and youth. The Colombian and the Suriname experiences appeared to be the most successful, followed by the Chilean and Ecuadorian initiatives. The Guatemalan experience was limited in this regard due to difficulties with the public health system.

*Access *refers to improvement in the ability of individuals to obtain required services at the right time and place. Despite the geographic isolation, the Suriname experience shows a remarkable level of access. The Chilean and the Colombian initiatives suggest increased access. The Ecuadorian and Guatemalan cases are somewhat more ambiguous in this regard. The mere existence of these initiatives stimulated attention to reducing barriers to access and increasing health resources for indigenous people but additional quantitative data is needed to determine the access issue more accurately.

The ability to provide uninterrupted, coordinated service across programs, practitioners, organizations and levels of service over time appears to have been enhanced by most of the cases studied. This *coordination and integration *is exemplary in the Colombian experience. The Suriname initiative shows a model of two separate organizations working very well in coordination. The Chilean case suggests a degree of progress in this regard. It is in the Ecuadorian and Guatemalan experiences where the level of integration in particular is quite limited.

Limited data has hindered the possibility of assessing *efficiency and flexibility *of the experiences, in the sense of achieving desired results with the most cost-effective use of resources as well as the degree to which the initiatives are flexible to new requirements. The fact that these experiences are essentially community-based indicates that they are more flexible than top-down health systems. Although in most cases there were suggestions of cost-effectiveness, more comprehensive and detailed data is needed.

In all five cases, *leadership *represented as the ability to initiate, spur, encourage, inspire and catalyze change was evident. This leadership took different forms, and was particularly strong in the Chilean and Colombian experiences, but was also present in the other cases.

The development of new and creative solutions that meet or surpass known standards is understood as being *innovative*. All five cases demonstrated, to varying degrees, that they meet this criterion.

To some extent, all cases can serve as a model for *replication *by others. In fact the Makewe experience in Chile spurred the Boroa initiative, as the success in Suriname's Kwamalasamutu case facilitated its replication in Pëlele Tëpu and elsewhere. However, each initiative is clearly context dependent, so only certain principles and organizational aspects are ultimately replicable.

All cases emerged as attempts to *resolve health and policy issues identified *by the indigenous communities. The organizations that emerged were having a significant influence on health policy at local and state levels. This was particularly evident in the cases of Chile and Colombia.

*Capacity for evaluation *refers to the capacity to measure outcomes to inform decision-making and assess the effectiveness of strategies and programs as well as client satisfaction within the best practice. However, there is a dearth of the data and information systems required for proper evaluations. The Suriname experience is the exception, because both clinics collect data on patient visits and make an effort to computerize these records. The Colombian case has potential since all services are managed by an indigenous insurance organization that must be accountable to government funders, but resources are not available to systematically record all health care transactions. Similarly, the Chilean initiative has more potential for evaluation given that data is collected on patient services at the local level. However, even these nascent information systems rarely document intercultural health contacts. In all cases, the state appears to have a negligible capacity or interest to create information systems that may evaluate not only intercultural health initiatives, but the publicly funded system itself.

## Discussion and conclusion

Traditional medicinal knowledge and healing practices of indigenous peoples throughout the world continue to play an important role in health care, both in parallel and in some cases in conjunction with western medicine. In Latin America, this situation exists in a context of larger socio-economic and political processes, including state controlled health systems, economic instability, constitutional and health reforms, and the marginalization of indigenous medicine and cultural identity [6,7]. Previously, research into traditional and contemporary health care practices among indigenous populations examined topics such as utilization patterns [8], relationships between community members and formal health services [9], and the impact of cultural conflict or change on health status [10]. However, in light of recent indigenous rights movements and the rapid expansion of pharmaceutical developments to exploit plants and local expertise, perspectives on this complex set of issues have become highly politicized. Many researchers are presently examining the state of traditional medicine's regulation throughout South America (i.e. integration, coexistence and tolerance) [11] and the need for community participation in health initiatives that are linked with indigenous rights and empowerment [12,13].

Another approach taken to study this development is that of intercultural health initiatives, which are designed to incorporate traditional and western medical practices within health care systems that are preferably led and managed through indigenous organizations. Using case study data, this paper reported on different models of intercultural health projects currently underway in Chile, Colombia, Ecuador, Guatemala, and Suriname.

Four main themes were comparatively analyzed: cultural, funding and management approaches to intercultural health service development; opportunities and benefits provided by intercultural initiatives; constraints and risks associated with the articulation of indigenous and western health systems; and an assessment of the impacts of intercultural health system development. As well, the cases were examined against best practice criteria seeking to assess the degree of successful development of the intercultural health initiatives. While space does not permit a thorough analysis of each topic, the breadth of our investigation speaks to the range of topics involved in intercultural health projects. The risks and benefits for indigenous populations and organizations are of particular significance, as they highlight the political tensions between state-run health systems and the largely marginalized practices of traditional healing. They are also related to the significant increase in community mobilization, inter-generational transmission of traditional knowledge, and the reclaiming of cultural integrity as a result of their participation. The plausibility of positive impact on health of these initiatives was also identified.

The study had a number of limitations. First, the study design sought to assess the cases against certain criteria but was not an effectiveness assessment of adequacy, plausibility or probability [14]. Second, information systems limitations of most case studies hindered the possibility of using reliable financial, epidemiological, and health utilization data for study purposes. Third, although significant fieldwork was done by local consultants prior to that of the principal investigators, the time spent by the latter collecting data in each intercultural case study was somewhat less than ideal.

The study identified a series of recommendations for policy makers at local, national and international levels. These include, the development of a culturally appropriate regulatory environment, de implementation of contractual models for promoting indigenous autonomy in health system development, supporting the shift from exclusively western health systems toward intercultural health programs and practices, supporting indigenous organizations and communities in the development of health programs, providing technical and financial support to develop information systems for monitoring, evaluation and research purposes, and fostering the exchange of ideas and models across Latin American countries and between North and South America. Among recommendations specific to research, the agenda should be to prioritize effectiveness studies.

## Competing interests

The author(s) declare that they have no competing interests.

## Authors' contributions

Javier Mignone, Judith Bartlett and John O'Neil equally conceived the study, conducted field work and wrote the main report of the study. They also equally contributed to the manuscript. Treena Orchard contributed to the study by assisting with the analysis of data, by writing sections of the initial study report and contributing to writing the manuscript.
